# Opportunities and Challenges for Microbial Synthesis of Fatty Acid-Derived Chemicals (FACs)

**DOI:** 10.3389/fbioe.2021.613322

**Published:** 2021-01-26

**Authors:** Yilan Liu, Mauricio Garcia Benitez, Jinjin Chen, Emma Harrison, Anna N. Khusnutdinova, Radhakrishnan Mahadevan

**Affiliations:** ^1^Department of Chemical Engineering and Applied Chemistry, University of Toronto, Toronto, ON, Canada; ^2^Institute of Biomedical Engineering, University of Toronto, Toronto, ON, Canada

**Keywords:** fatty acid-derived chemicals, microbial chassis, systems engineering, model-assisted design, review

## Abstract

Global warming and uneven distribution of fossil fuels worldwide concerns have spurred the development of alternative, renewable, sustainable, and environmentally friendly resources. From an engineering perspective, biosynthesis of fatty acid-derived chemicals (FACs) is an attractive and promising solution to produce chemicals from abundant renewable feedstocks and carbon dioxide in microbial chassis. However, several factors limit the viability of this process. This review first summarizes the types of FACs and their widely applications. Next, we take a deep look into the microbial platform to produce FACs, give an outlook for the platform development. Then we discuss the bottlenecks in metabolic pathways and supply possible solutions correspondingly. Finally, we highlight the most recent advances in the fast-growing model-based strain design for FACs biosynthesis.

## Introduction

Increasing consumption of petroleum-derived products leads to increasing atmospheric carbon dioxide (CO_2_) levels and global warming (Sperry et al., 2019). Furthermore, the uneven distribution and unsustainability of fossil resources have motivated engineers to seek alternative sustainable solutions (Raslavičius et al., 2014; Chen et al., 2020). Compared with the traditional strategies to convert plant oils and animal fats into biodiesel, microbial synthesis of fuels, and chemicals presents several advantages. Firstly, feedstocks can be shifted from edible plant oils and animal fats to non-edible biomass feedstocks, especially CO_2_. Secondly, due to the flexibility of pathways in microbial chassis, a large diversity of bioproducts can be produced in microbial cell factories. Among these bioproducts, fatty acid-derived chemicals (FACs) have attracted significant attention, because fatty acids (FAs) are essential metabolites in all organisms. FAs and their biosynthetic/catabolic intermediates can be used as precursors for a large diversity of FACs, which have an unprecedented wide application range: biofuels, pharmaceuticals, feed additives, and others. Thirdly, bioproducts are green alternatives to petroleum-based fuels, given the capacity of net-zero greenhouse gas emissions. Microbial chassis must be extensively designed and engineered to produce FACs at high titer, rate and yield from various substrates. Recent successes in model-based strain design have speed-up the Design-Build-Test-Learn (DBTL) cycle in metabolic engineering (Carbonell et al., 2018; Hamedirad et al., 2019; Opgenorth et al., 2019). Although FACs biosynthesis has been reviewed from different angles (Marella et al., 2018; Liu and Li, 2020), the purpose of this review is to update the most recent advances in this fast-developing field, with an emphasis on possible synthetic microbial chassis and computational modeling for biosynthesis of FACs.

## Types and Applications of Fatty Acid-Derived Chemicals

**FIGURE 1:**
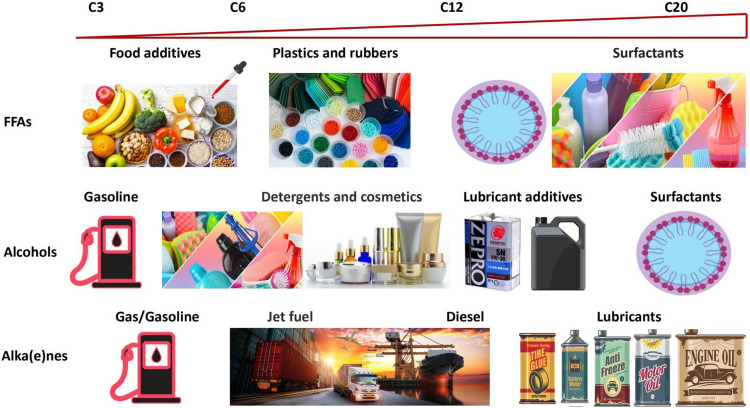
Overview of types and applications of FACs. FACs have a wide application range based on their various chemical properties caused by different end groups and chain lengths.

With accelerating concerns over climate change and the environmental impact of conventional production methods, interest in the renewable microbial production of chemicals have grown (Liu and Nielsen, 2019; Cho et al., 2020; Li M. et al., 2020; Sgobba and Wendisch, 2020; Wu et al., 2020). Among these chemicals, FACs are of particular interest due to their various applications in biofuels, detergents, medicines, industrial lubricants, bioplastics, emulsifiers, food and feed additives, and others (Supplementary Table 1; Richardson and Mcallister, 1945; Geller and Goodrum, 2004; Bellou et al., 2016; Jiang W. et al., 2018; Li G. et al., 2020; Verma et al., 2020; Zerhusen et al., 2020). Different end groups and lengths of FACs lead to different physical and chemical properties, which in turn lead to different practical applications (Figure 1). In general, FACs can be mainly classified into free fatty acids (FFA), fatty alcohols, alka(e)nes, and fatty acid esters (FAEs) (Steen et al., 2010). Most naturally occurring FACs have an unbranched chain within the range of C3 to C28. Based on the chain length, they are generally classified into short-chain (≤ 6), medium-chain (7–12), long-chain (13–20), and very-long-chain (> 20) (Schönfeld and Wojtczak, 2016). However, the definitions can vary from one study to another (Rodriguez-Moya and Gonzalez, 2015).

Fatty acids, one of the most studied FACs, are n-alkyl carboxylic acids with an aliphatic chain. Short-chain fatty acids play an important role in human health. For example, butyric acid can be used in food and pharmaceutical industries (Jiang L. et al., 2018). Most free FFAs are converted into biofuels, or consumer products (Leber et al., 2015; Marella et al., 2018; Sathesh-Prabu et al., 2019). Fatty alcohols have hydroxyl groups attached to the aliphatic chain. Short-chain alcohols, such as 1-propanol and 1-butanol, can be used as gasoline or fuel additives (Guo et al., 2019; Zhou et al., 2019). Alcohols with a chain length of C8–C10 are important materials to produce detergents, lubricants, cosmetics, pharmaceuticals, and plastics. Those in C12–C14 range are used as lubricant additives, and those in C16–C18 range are used for drug delivery and defoamers (Dong and Mumper, 2006; Zheng et al., 2012). Among these, C11–C14 alcohols, the key intermediates for surfactants production, represent 55% of the market share (Fillet and Adrio, 2016). Alkanes are saturated hydrocarbons with the general chemical formula C_*n*_H_2*n*+2_, while alkenes are unsaturated hydrocarbons containing at least one C–C double bond. Alka(e)nes are an important class of FACs because of their high similarity to petroleum-derived fuels. Depending on the chain length, alka(e)nes have different applications, including drop-in fuels in gasoline (C3–C9), jet fuel (C8–C16), diesel (C10–C18), and lubricants (C16–C30) (Kang et al., 2017). In addition to the FACs mentioned above, there are other important fatty acid-based chemicals, such as fatty acid alkyl esters (FAAEs) and branched FACs that are used for certain applications due to their specific properties (Röttig et al., 2010; Ngo et al., 2013; Gupta et al., 2015; Teo et al., 2015; Bentley et al., 2016; Jiang et al., 2017; Shrestha and Yamamoto, 2018; Singh and Choudhury, 2018). For examples, branched fatty alcohol 4-methyl-pentanol is a common brake fluids (Shrestha and Yamamoto, 2018; Supplementary Table 1).

## Microbial Chassis for Biosynthesis of FACs

**FIGURE 2:**
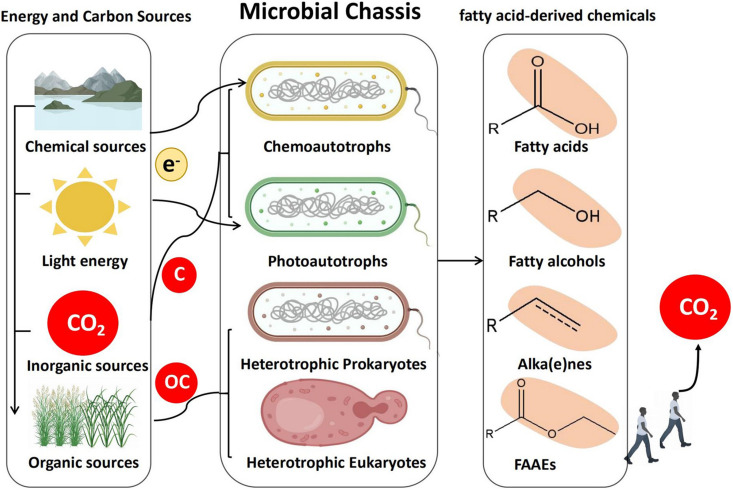
Strategies for production of FACs in microbial chassis. Autotrophs and heterotrophs synthesize FACs from CO_2_ and organic carbon (OC), respectively. Either platforms have their own advantages and limitations. Autotroph-heterotroph consortia represent a promising platform for microbial production of FACs.

Theoretically speaking, any microorganism can be used as a microbial chassis for biosynthesis of FACs, because fatty acid metabolic pathways exist in all living cells. Currently, most researches are devoted to model organisms such as *Escherichia coli* and *Saccharomyces cerevisiae* (Liu et al., 2016; Hu et al., 2019; Kim and Park, 2019; Yang et al., 2020). In our opinion, there are some other promising microorganisms, which have not been explored. In this review, microorganisms are classified into four groups including chemoautotroph, photoautotroph, heterotrophic prokaryotes, and heterotrophic eukaryotes. Their advantages, disadvantages, and the promising synthetic community strategy for microbial production of FACs will be discussed in detail (Figure 2).

Chemoautotrophs are organisms that can synthesize their own organic molecules through the fixation of carbon dioxide. Energy required for this process comes from the oxidation of inorganic molecules such as iron, sulfur, or magnesium (Thakur et al., 2018). Though research efforts on chemoautotrophic bacteria have started to gain attention, the application of chemoautotrophs at an industrial scale is still challenging, due to their slow growth pattern and the limited applicable genetic engineering tools. *Cupriavidus necator*, which has one of the highest growth rates among natural autotrophic bacteria, was successfully used to produce FACs from CO_2_. However, the autotrophic production level of FACs was much lower compared to heterotrophic production on fructose (Crépin et al., 2016). Another chemolithotrophic oleaginous bacterium, *Rhodococcus opacus*, was engineered to produce fatty acids and fuels as high as 50.2 g/L, however this was carried out under heterotrophic condition with glucose as carbon source (Kim et al., 2019). Recently some chemoautotrophs were observed to utilize electricity as energy resources for biosynthesis, which make them promising microbial chassis (Geelhoed and Stams, 2011). Photoautotrophic microorganisms are cells that capture light energy to fix carbon. Among these microorganisms, cyanobacteria are the most studied because it is easy to genetically modify (Liu et al., 2011; Tan et al., 2011; Wang et al., 2013), and biosynthesis of FACs has already been proven feasible in them (Liu et al., 2011; Eungrasamee et al., 2019). For examples, an important Omega−3 fatty acid was produced by overexpression of desaturase *desA* and *desB* in *Synechococcus* sp. PCC 7002 (Santos-Merino et al., 2018), and fatty alcohols were also successfully produced in photosynthesis-driven cyanobacteria (Tan et al., 2011; Yunus and Jones, 2018). By overexpressing acyl-acyl carrier protein reductase (AAR) and aldehyde decarbonylase (AD), metabolically engineered cyanobacterium, *Nostoc punctiforme*, produced alkanes at levels up to 12% of their cell dry weight (Peramuna et al., 2015). In the case of the heterotrophs, both heterotrophic prokaryotes and eukaryotes have been widely used for FACs biosynthesis (Wang et al., 2010; Rutter and Rao, 2016; Wu et al., 2017; Xin et al., 2017; McNeil and Stuart, 2018; Zhou et al., 2018; Wu et al., 2019). *E. coli*, the most commonly used heterotrophic prokaryote, was engineered to produce free fatty acids at a titer of up to 21.5 g/L (Xiao et al., 2016). The most commonly used heterotrophic eukaryote, *Saccharomyces cerevisiae* was designed and engineered to produce 33.4 g/L of extracellular free fatty acids (Yu et al., 2018). Some oleaginous heterotrophic eukaryotes, such as *Yarrowia lipolytica* and *Aureobasidium pullulans* show great potential for FACs biosynthesis, since just with simple adjustment they can reach much higher titers than the engineered *S. cerevisiae* (Xu et al., 2016; Xin et al., 2017). Although these microbial platforms have their own advantages, they also have their own limitations. For example, autotrophs can synthesize FACs from CO_2_ via using solar, chemical and electric energy. Due to the abundance of CO_2_ in the atmosphere and its role in driving global climate change, CO_2_-assimilating microbes represent a unique and promising type of microbial chassis for FACs biosynthesis. However, autotrophs normally have limited growth rates and genetic engineering tools, resulting in difficulties to engineer metabolic pathways to produce specific FACs. Comparatively, many genetic engineering tools have been developed for fast-growing and metabolically versatile heterotrophs. But using organic carbon substrates makes them less environmental friendliness. Therefore, a platform combining different types of microorganism is required for more economical, environmental, and efficient microbial factories. Fortunately, recent advances in synthetic biology have made synthetic microbial communities possible (Johns et al., 2016; Tang, 2019; Liu et al., 2020). There are microalgae-microalgae, microalgae and bacteria, microalgae and molds communities constructed for FACs production (Magdouli et al., 2016). Ideally, a microbial community system could use autotrophs to fix CO_2_ from the atmosphere and subsequently transfer the organic products to heterotrophs for FACs biosynthesis. One such system has already been reported, a *Synechococcus elongates-Pseudomonas putida* consortium was constructed to produce bioplastic (PHA, polyhydroxyakanotate) (García-Jiménez et al., 2018). Another microalgae-yeast co-culture was isolated from wastewater and identified to contain a number of microalgae and yeast species, which was also successfully used for fatty acid methyl esters production (Suastes-Rivas et al., 2019). From our perspective, these autotroph-heterotroph communities have the potential to produce specific FACs from CO_2_. However, the slow rates of CO_2_ fixation in autotrophs seriously affect their practical applications. We believe that direct evolution of these synthetic communities could be a promising solution to overcome this limitation (Chang et al., 2020).

## Systems Engineering Strategies for Biosynthetic Pathway Optimization

The metabolic pathway for FACs biosynthesis can be broadly divided into three steps: initiation, elongation and termination. It starts with the conversion of feed materials to the universal precursor acetyl-CoA through various conversion pathways. The most common pathway for the synthesis of acetyl-CoA is through glycolysis, which converts glucose into pyruvate, then can be decarboxylated to produce acetyl-CoA. However, the decarboxylation of pyruvate loses a carbon equivalent, thus limits the theoretical carbon yield, and constrains the commercialization potential. Fortunately, a non-oxidative pathway was built up to produce stoichiometric amounts of acetyl-CoA from hexose, pentose and triose phosphates without carbon loss (Bogorad et al., 2013). Another intriguing alternative to sugars is the potential to produce acetyl-CoA from one-carbon resources such as CO_2_ and formate (Lu et al., 2019). After external carbon sources being converted into acetyl-CoA, it can be directly used as initiation blocks or transformed to propionyl-CoA, acetyl-acyl carrier protein (ACP), and propionyl-ACP for initiation. The initiation pattern determines the odd or even carbon chain of the produced FACs (Supplementary Figure 1; Dellomonaco et al., 2011; Park et al., 2020; Zhang et al., 2020).

**FIGURE 3:**
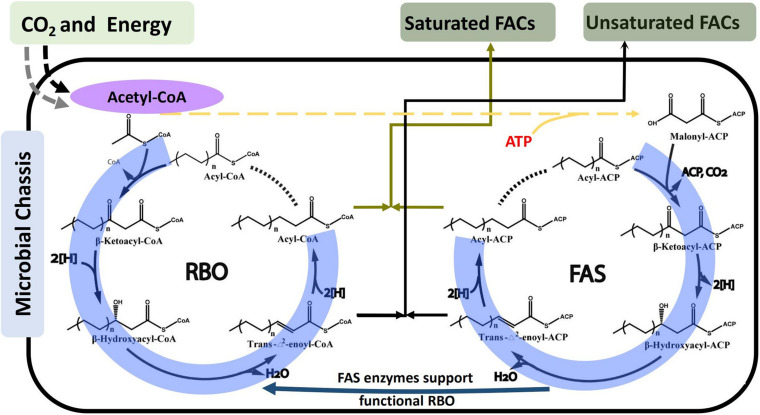
Schematic of metabolic pathways for FACs biosynthesis via Reverse Beta Oxidation (RBO) and Fatty Acid Synthesis (FAS). The entry of CO_2_ and energy to acetyl-CoA depends on the type of microbial chassis selected, as shown in Figure 2. The catalytic reaction cycles for both RBO and FAS include the same four serial steps: condensation, reduction, dehydration, and reduction. The major difference is that RBO employs a non-decarboxylative condensation mechanism to incorporate acetyl-CoA while FAS utilizes a decarboxylative condensation mechanism to incorporate malonyl-ACP.

In terms of elongation, fatty acid synthesis (FAS) and reverse beta-oxidation (RBO) pathways are the two identified routes for FACs biosynthesis. Although the four serial steps including condensation, reduction, dehydration, and reduction are similar in both FAS and RBO pathways (Figure 3), the iterative feeding strategies are different. In FAS pathway, acetyl-CoA was transferred into malonyl-ACP before being fed into the elongation cycle, while acetyl-CoA was directly fed into the elongation cycle in RBO pathway (Figure 3). FAS has been most widely studied and engineered to produce free fatty acids, alcohols, esters, and alkanes (Liu et al., 2016, 2018; Wenning et al., 2017; Yunus and Jones, 2018). However, RBO is widely accepted as the promising pathway for several reasons. Firstly, one ATP will be saved via the RBO pathway, as acetyl-CoA can be directly fed into the elongation cycle, while for elongation in FAS, acetyl-CoA must first be converted to malonyl-CoA via an ATP-consuming acetyl-CoA carboxylase. Secondly, most reductases from the FAS pathway have been shown to prefer NADPH as cofactors (Ratledge, 2004; Handke et al., 2011; Javidpour et al., 2014). In contrast, reductases from the RBO routes are mostly NADH-dependent (Lian and Zhao, 2014; Sheppard et al., 2016; Kim and Gonzalez, 2018). Since it has been demonstrated that cell has relatively high NADH/NAD^+^ ratio under anaerobic condition (De Graef et al., 1999), RBO will be benefited in the anaerobic biosynthesis of FACs. Thirdly, RBO pathway is dependent on the universal CoA molecule, while FAS pathway is dependent on organism specific A, making RBO pathway more transferable in target microorganisms. Recently, it was reported that with the exception of condensation step, the remaining enzymes for other steps in FAS pathway: 3-ketoacyl-ACP reductase (*FabG*), 3-hydroxyacylACP dehydratase (*FabZ*), and enoyl-ACP reductase identified (*FabI*) can carry out similar conversions as in RBO in *E. coli* (Vick et al., 2015; Clomburg et al., 2018). It was reported that some of these enzymes show preference for acyl-ACP intermediates, such as *fabZ* from *E. coli* (Tsuge et al., 2003). These findings present both opportunities and challenges. On one hand, it offers the potential to employ FAS enzymes on various acyl-CoA intermediates, which can greatly expand the range of FACs produced by RBO. On the other hand, it will lead to promiscuous activity and make it difficult to produce specific FACs, as intracellular substrates will be automatically used by the endogenous FAS enzymes, which results in impure and unwanted products. Though both of FAS and RBO pathways can be used to produce FACs with different chain lengths, FAS pathway is preferred for long-chain FACs production, because it naturally has high efficiency; while for short-chain FACs, the RBO pathway is favored because it is easier to control product lengths than FAS pathway (Sheppard et al., 2016).

The termination step, which releases fatty acyl-CoA or fatty acyl-ACP from the elongation cycle, is the most important and widely investigated step, as it determines the types of FACs produced by microbial cell factories. For each type of FACs, there are multiple options for termination. For example, fatty alkenes and fatty alcohols can be generated from fatty acids, fatty acyl-ACPs and fatty acyl-CoAs (Liu et al., 2016; Liu and Li, 2020). Alkanes can be converted from fatty aldehydes by aldehyde decarbonylase or from fatty acids by photodecarboxylase (Eser et al., 2011; Sorigué et al., 2017). Even though numerous terminal pathway options have been found, it is still the major bottleneck for FACs biosynthesis for the following reasons: First, production of FACs other than FFAs is not efficient. According to our knowledge, the highest titer of mixed long chain FFAs (C14–C22) is 50.2 g/L using an oleaginous bacterium *Rhodococcus opacus* PD630 (Kim et al., 2019), while the highest titers of fatty alcohols and alka(e)nes is 12.5 and 2.54 g/L, respectively (Fatma et al., 2018). Considering the same upstream pathway, the low titers of fatty alcohols and alka(e)nes are perhaps caused by the low efficiency of enzymes in the termination step. Second, enzymes in terminal step naturally prefer longer chain substrates. Although great efforts have been made for short chain substrates, the problem is far from being resolved (Khara et al., 2013; Gajewski et al., 2017). Hence, screening and engineering of enzymes that prefer short chain substrates should be an important area of research.

## Model-Assisted Design for Biosynthesis of FACs

**FIGURE 4:**
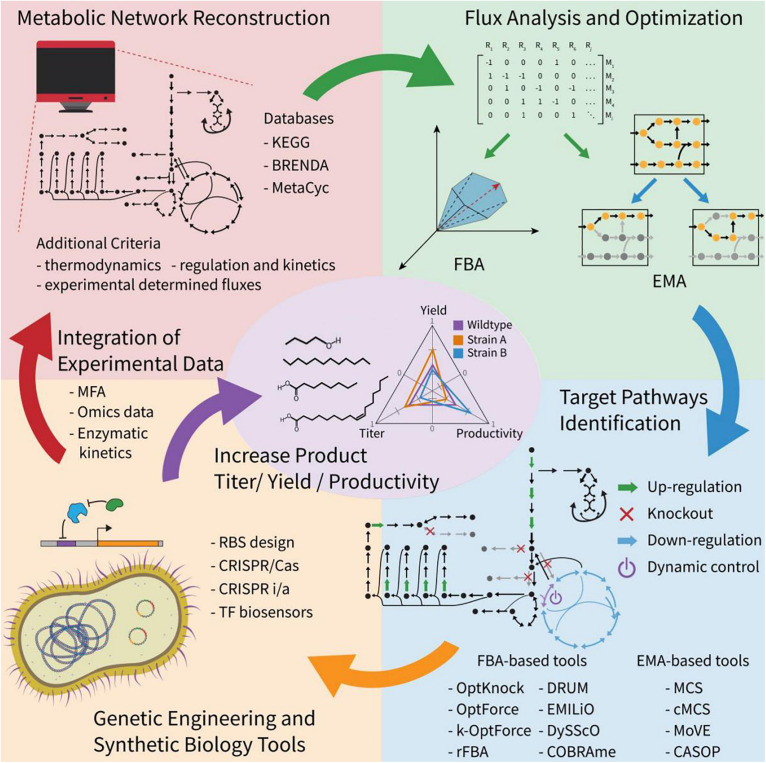
Overview of computational model-assisted design algorithms. Computational methods have been developed to identify pathways to enable production of desired compounds using known or *de novo* metabolic pathways. Additional host metabolic strategies, including up/down-regulation, knockout, and dynamic control can be designed using a variety of constraint-based modeling algorithms.

Model-assisted design has shown to be successful in metabolic engineering (Teusink and Smid, 2006; Fatma et al., 2018; Ferreira et al., 2019; Das et al., 2020; Figure 4). Increasing information in databases, such as KEGG, BioCyc, BRENDA, MetRxn, and SEED (Shin et al., 2013; Long et al., 2015; Delépine et al., 2018; Choi et al., 2019), makes it possible to develop organism-specific reaction networks, *de novo* pathway predictions and even retrosynthetic design of metabolic pathways for non-natural chemicals (Medema et al., 2012; Tabei et al., 2016; Biz et al., 2019; Garcia and Trinh, 2019a). Model-assisted design facilitates efficient Design-Build-Test-Learn (DBTL) cycle, avoiding costly trial and error approaches (Long et al., 2015; Choi et al., 2019). There are two basic metabolic analysis algorithms for model-guided design in metabolic engineering: Flux balance analysis (FBA) and Elementary mode analysis (EMA) (Mahadevan et al., 2002; Klamt and Gilles, 2004; Machado and Herrgård, 2015). FBA uses linear optimization to find a set of reaction fluxes that satisfy both an objective function and a set of constraints limiting the solution space of the network representing a given growth condition (Orth et al., 2010). EMA calculates all the solutions with minimal support in the network that satisfy the steady state and other constraints (Trinh et al., 2009). Each solution in EMA is called an elementary mode (EM) and describes the topology of the metabolic network, which is useful in determining its properties and for rational design (Trinh et al., 2009). Most computational tools are derived from EMA or FBA for more specific purposes (Rodrigo et al., 2008; Trinh et al., 2009; Campodonico et al., 2014; Long et al., 2015; Garcia and Trinh, 2019b, 2020; Jiang et al., 2020).

Methods based on FBA, such as OptKnock, OptStrain, OptForce, dFBA, DySScO DynamicME, and COBRAme have been developed for strain engineering purposes, to identify a set of genetic interventions to increase the production of target compounds (Mahadevan et al., 2002; Burgard et al., 2003; Pharkya et al., 2004; Ranganathan et al., 2010; Zhuang et al., 2013; Lloyd et al., 2018; Yang et al., 2019). The two most used design programs based on FBA are OptKnock and OptForce. OptKnock is the first bi-level optimization framework for strain design, which can identify optimal reaction deletion strategies that couple cellular growth with the production of a target metabolite (Burgard et al., 2003). A successful case of OptKnock algorithm application is a growth-coupled strategy designed for biofuel production in *Synechocystis*, and it shows that lowering the ATP/NADPH ratio in the cell is a promising strategy for biosynthesis of fatty alcohols and alkanes (Shabestary and Hudson, 2016). OptForce is a framework predicting genetic interventions such as overexpression and repression based on the comparison of an initial metabolic status and the desired overproduction goal (Ranganathan et al., 2010). It can prioritize the interventions according to their effects on the increment of the production, making it possible to start with the modifications that would have higher impact on the process. The OptForce algorithm has been successfully used for strategy design in *E. coli* for fatty acids production. Moreover, it can predict less intuitive interventions, such as the redirection of the flux through the Entner-Doudoroff pathway to produce NADPH and induce a growth arrest limiting the ATP production (Ranganathan et al., 2012; Tee et al., 2014). Recently, another study applied OptForce for the production of octanoic acid, achieving high selectivity (>70%) and an extracellular concentration up to 1 g/L of free octanoic acid in minimal medium via fed-batch culture (Liu et al., 2018). A metabolic model was constructed for long-chain alkane and alcohol production based on FBA analysis, and the engineered strain produced the maximum titers of hydrocarbons (Fatma et al., 2018). Besides, recently breakthroughs have been made in visualizing genome-scale metabolic flux networks, which improved understanding of the predicted solutions (Chazalviel et al., 2018; Hari and Lobo, 2020).

EMA-based tools have been used for strain engineering by applying the concept of minimal cut sets (MCS) (Klamt and Gilles, 2004). Constrained MCS (cMCS) was developed to remove limitations in MCS, where many solutions also eliminated growth (Hädicke and Klamt, 2011). Using cMCS, researchers identified sets of reactions to eliminate and enhanced the production of ethanol and isobutanol in *Clostridium thermocellum* and cyanobacteria, respectively (Erdrich et al., 2014; Thompson and Trinh, 2017). There has been interest in dynamic control strategies, which can dynamically regulate of flux through metabolite sensor, inducer, temperature, light and cell density (Lalwani et al., 2018; Liu et al). These provide the option to prioritize growth or production in a two-stage process, which can lead to higher yields, productivities and titers of FACs (Zhang et al., 2012; Lalwani et al., 2018; Raj et al., 2020). To accelerate the strain engineering process for enhanced chemical production, MODCELL and MODCELL 2 frameworks were developed for rapid generation of optimal production strains by systematically assembling a modular cell with an exchangeable production module (Trinh et al., 2015; Garcia and Trinh, 2019c). Moreover, MoVE, a newly developed tool based on MCS, can identify genetic interventions that allow the transition between growth and production states for dynamic control of the metabolism (Venayak et al., 2018).

Once engineering strategies are obtained from computational modeling, there are plenty of synthetic biology tools available to implement the suggested metabolic engineering interventions. For instance, CRISPR-based technologies make it possible to perform multiple knockouts, inhibitions, or activations of designed sets (Behler et al., 2018; Kaczmarzyk et al., 2018; Reis et al., 2019). Significant improvements have been achieved in FACs biosynthesis using model-based strain design strategies (Matsuda et al., 2011; Shabestary and Hudson, 2016; Fatma et al., 2018; Yu et al., 2018), however, there remain challenges to be addressed in future studies. For example, there is still a lack of methods to integrate large amounts of data into genome-scale models and provide user-friendly tools that allow users with no programming experience to exploit the potential of genome-scale metabolic models for rational design. In conclusion, model-based strain engineering is still in an early stage and its application has been limited to few chemical targets and tools. We expect that the development of novel user-friendly computational models can enable increased adoption of such tools for various types of FACs production.

## Conclusion

The ongoing reliance on fossil fuels of human society is driving elevated atmospheric CO_2_ and increasing global temperatures, thereby escalating the risk of widespread environmental disasters in the near future. We anticipate that microbial synthesis of products from CO_2_, which can provide chemicals with near-zero net greenhouse gas emissions, will play as a game-changer in the future (Ediger, 2019). Great progress has been made in the areas of enzyme engineering, metabolic engineering, and model-assisted engineering to assist microbial production of FACs (Cao et al., 2016; Herman and Zhang, 2016; Kim et al., 2016; Zhou et al., 2016; Fatma et al., 2018; Marella et al., 2018; Kim and Park, 2019; Liu and Nielsen, 2019; Lynch et al., 2019). However, the present-day microbial cell factories still have major challenges to overcome, such as controlling the length and types of released FACs and improving the conversion efficiency via RBO. We expect that directed enzyme evolution and rational enzyme engineering will contribute to the production of target FACs through the RBO pathway. Recently, there are some machine learning-based algorithms developed for computational protein design, which can also be used in enzyme engineering (Masso and Vaisman, 2008; Fang, 2019; Zu Belzen et al., 2019). In addition, new methods for design and build of synthetic microorganism communities can contribute to the construction of novel microbial platforms, which combine carbon-fixing autotrophs with heterotrophs for efficient FACs biosynthesis with net-zero greenhouse gas emissions.

## Author Contributions

YL and RM conceived of the idea. YL, MB, JC, EH, and AK wrote the manuscript. YL, JC, EH, MB, and RM contributed to revising. All authors contributed to the article and approved the submitted version.

## Conflict of Interest

The authors declare that the research was conducted in the absence of any commercial or financial relationships that could be construed as a potential conflict of interest.

## Supplementary Material

Supplementary Figure 1 Initiation and feed in blocks in Reverse Beta Oxidation (RBO) **(A)** and Fatty Acid Synthesis (FAS) **(B)** pathways. Each turn of the FAS and RBO cycles leads to the addition of two carbons through incorporation of malonyl-ACP or acetyl-CoA, respectively. Consequently, the initiation blocks control over whether the produced FACs have odd or even carbon chains.
