# Supplementary material for: Opportunities and Challenges for Microbial Synthesis of Fatty Acid-Derived Chemicals (FACs)
Source: Front Bioeng Biotechnol. 2021 Jan 26;9:613322. doi: 10.3389/fbioe.2021.613322 (PMC7870715; doi:10.3389/fbioe.2021.613322)
Supplement: Supplementary Figure 1 — Initiation and feed in blocks in Reverse Beta Oxidation (RBO) (A) and Fatty Acid Synthesis (FAS) (B) pathways. Each turn of the FAS and RBO cycles leads to the addition of two carbons through incorporation of malonyl-ACP or acetyl-CoA, respectively. Consequently, the initiation blocks control over whether the produced FACs have odd or even carbon chains. [file Table_1.DOCX]

Supplementary Material

# Supplementary Figures and Tables


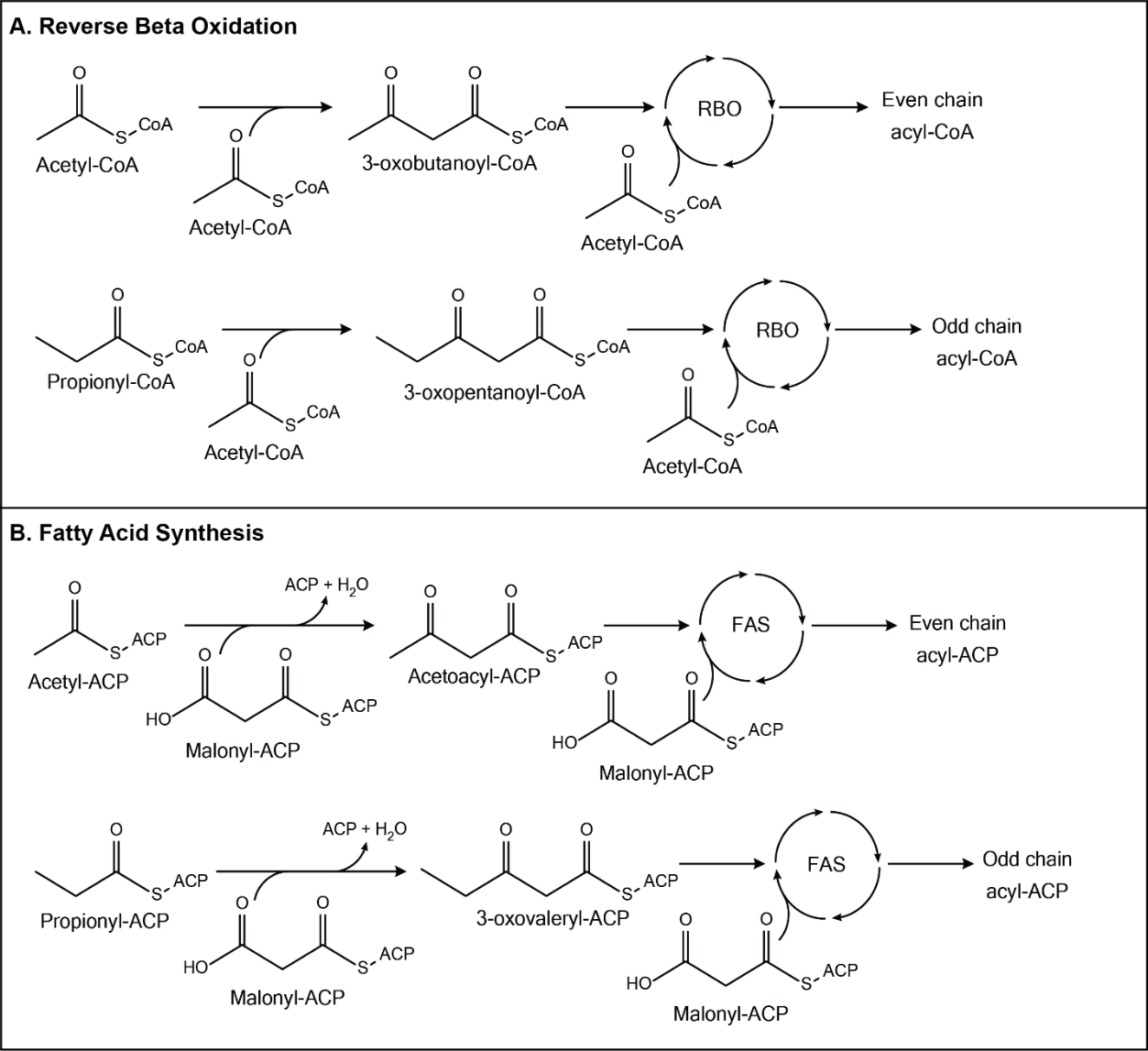


**Supplementary Figure 1** Initiation and feed in blocks in Reverse Beta Oxidation (RBO) (A) and Fatty Acid Synthesis (FAS) (B) pathways. Each turn of the FAS and RBO cycles leads to the addition of two carbons through incorporation of malonyl-ACP or acetyl-CoA, respectively. Consequently, the initiation blocks control over whether the produced FACs have odd or even carbon chains.

**Supplementary Table 1. Production of FACs and their applications.**

| Category | Product | Titer (g/l) | Yield (g/g) | Host | Application | Ref |
| --- | --- | --- | --- | --- | --- | --- |
| Free fatty acids (FFAs) | Butyric acid | 60.4 | 0.38 | *Clostridium tyrobutyricum* | Mainly for cellulose acetate butyrate plastics, also as the therapeutic agent | (Huang et al., 2011) |
|  | Hexanoic acid | 32 | 0.34 | *Clostridium sp.BS-1* | Medicine, food additives, rubber, perfumes, dyes, lubricating grease | (Jeon et al., 2013) |
|  | C6-C10 | 3.8 | NA | *Escherichia coli* | Precursor for industrial chemicals and diesel‐like biofuels | (Wu et al., 2019) |
|  | C10-C18 | 10.4 | 0.047 | *Saccharomyces cerevisiae* | Mainly as surfactants, also as precursor of biokerosene | (Rigouin et al., 2017) |
|  | C14-C22 | 50.2 | 0.259 | *Rhodococcus opacus* | Biokerosene | (Kim et al., 2019) |
| Fatty alcohols | 1-Propanol | 0.72 | NA | *Escherichia coli* | Antiseptic solutions and precursor of biofuel | (Siebert and Wendisch, 2015) |
|  | 1-Butanol | 16.9 | 0.41 | *Clostridium saccharoperbutylacetonicum* | Latex surface coatings, enamels, and lacquers | (Thang et al., 2010) |
|  | C6-C10 | 0.3 | NA | *Escherichia coli* | Surface active agents or detergents | (Kim et al., 2015) |
|  | C12/14 | 1.55 | 0.13 | *Escherichia coli* | Detergents, emulsifiers, lubricants, and cosmetics | (Youngquist et al., 2013) |
|  | C16-C18 | 8 | 0.04 | *Rhodosporidium toruloides* | Surfactants, detergents, emollients and thickeners, polymerization agents, emollients and thickeners and diesel-like biofuels | (Fillet et al., 2015) |
|  | C12-18 | 12.5 | 0.28 | *Escherichia coli* | Detergents, surfactants, lubricants, additives for gasoline | (Fatma et al., 2018) |
| Alkanes/  Alkenes | Propane | 0.032 | NA | *Escherichia coli* | Polymer precursors, gasoline components and diluent | (Kallio et al., 2014) |
|  | Pentane | 0.049 | NA | *Yarrowia lipolytica* | Gasoline main component | (Blazeck et al., 2013) |
|  | C8-16 | 0.580 | NA | *Escherichia coli* | Jet fuel | (Choi and Lee, 2013) |
|  | C13-C17 | 2.54 | 0.25 | *Escherichia coli* | Diesel | (Fatma et al., 2018) |
| Fatty acid alkyl esters (FAAE) | Fatty acid ethyl esters (FAEE) | 21.3 | 0.086 | *Rhodococcus opacus* | Biodiesel | (Kim et al., 2019) |
|  | Fatty acid methyl esters (FAME) | 1.53 | NA | *Scenedesmus abundans* | Biodiesel | (Mahesh et al., 2019) |
| Branched-chain FACs | C12-C20 | 0.18 | NA | *Escherichia coli* | Cosmetics, lubricants, fuel additives, surfactants, coatings, soaps, and body wash | (Bentley et al., 2016) |
|  | 4-methyl-pentanol | 0.19 | NA | *Escherichia coli* | Gasoline replacements | (Sheppard et al., 2014) |
|  | C12-C18 Branched-chain fatty alcohols | 0.35 | NA | *Escherichia coli* | Cosmetics and fragrances | (Jiang et al., 2017) |

Supplementary References

Bentley, G.J., Jiang, W., Guamán, L.P., Xiao, Y., and Zhang, F. (2016). Engineering Escherichia coli to produce branched-chain fatty acids in high percentages. *Metabolic engineering* 38**,** 148-158.

Blazeck, J., Liu, L., Knight, R., and Alper, H.S. (2013). Heterologous production of pentane in the oleaginous yeast Yarrowia lipolytica. *Journal of biotechnology* 165**,** 184-194.

Choi, Y.J., and Lee, S.Y. (2013). Microbial production of short-chain alkanes. *Nature* 502**,** 571-574.

Fatma, Z., Hartman, H., Poolman, M.G., Fell, D.A., Srivastava, S., Shakeel, T., and Yazdani, S.S. (2018). Model-assisted metabolic engineering of Escherichia coli for long chain alkane and alcohol production. *Metabolic engineering* 46**,** 1-12.

Fillet, S., Gibert, J., Suárez, B., Lara, A., Ronchel, C., and Adrio, J.L. (2015). Fatty alcohols production by oleaginous yeast. *Journal of industrial microbiology & biotechnology* 42**,** 1463-1472.

Huang, J., Cai, J., Wang, J., Zhu, X., Huang, L., Yang, S.-T., and Xu, Z. (2011). Efficient production of butyric acid from Jerusalem artichoke by immobilized Clostridium tyrobutyricum in a fibrous-bed bioreactor. *Bioresource Technology* 102**,** 3923-3926.

Jeon, B.S., Moon, C., Kim, B.-C., Kim, H., Um, Y., and Sang, B.-I. (2013). In situ extractive fermentation for the production of hexanoic acid from galactitol by Clostridium sp. BS-1. *Enzyme and microbial technology* 53**,** 143-151.

Jiang, W., Qiao, J.B., Bentley, G.J., Liu, D., and Zhang, F. (2017). Modular pathway engineering for the microbial production of branched-chain fatty alcohols. *Biotechnology for biofuels* 10**,** 244.

Kallio, P., Pásztor, A., Thiel, K., Akhtar, M.K., and Jones, P.R. (2014). An engineered pathway for the biosynthesis of renewable propane. *Nature communications* 5**,** 1-8.

Kim, H.M., Chae, T.U., Choi, S.Y., Kim, W.J., and Lee, S.Y. (2019). Engineering of an oleaginous bacterium for the production of fatty acids and fuels. *Nature Chemical Biology* 15**,** 721-729.

Kim, S., Clomburg, J.M., and Gonzalez, R. (2015). Synthesis of medium-chain length (C6–C10) fuels and chemicals via β-oxidation reversal in Escherichia coli. *Journal of industrial microbiology & biotechnology* 42**,** 465-475.

Mahesh, R., Naira, V.R., and Maiti, S.K. (2019). Concomitant production of fatty acid methyl ester (biodiesel) and exopolysaccharides using efficient harvesting technology in flat panel photobioreactor with special sparging system via Scenedesmus abundans. *Bioresource technology* 278**,** 231-241.

Rigouin, C., Gueroult, M., Croux, C., Dubois, G., Borsenberger, V., Barbe, S., Marty, A., Daboussi, F., André, I., and Bordes, F. (2017). Production of medium chain fatty acids by Yarrowia lipolytica: combining molecular design and TALEN to engineer the fatty acid synthase. *ACS Synthetic Biology* 6**,** 1870-1879.

Sheppard, M.J., Kunjapur, A.M., Wenck, S.J., and Prather, K.L. (2014). Retro-biosynthetic screening of a modular pathway design achieves selective route for microbial synthesis of 4-methyl-pentanol. *Nature communications* 5**,** 1-10.

Siebert, D., and Wendisch, V.F. (2015). Metabolic pathway engineering for production of 1, 2-propanediol and 1-propanol by Corynebacterium glutamicum. *Biotechnology for biofuels* 8**,** 91.

Thang, V.H., Kanda, K., and Kobayashi, G. (2010). Production of acetone–butanol–ethanol (ABE) in direct fermentation of cassava by Clostridium saccharoperbutylacetonicum N1-4. *Applied biochemistry and biotechnology* 161**,** 157-170.

Wu, J., Wang, Z., Zhang, X., Zhou, P., Xia, X., and Dong, M. (2019). Improving medium chain fatty acid production in Escherichia coli by multiple transporter engineering. *Food chemistry* 272**,** 628-634.

Youngquist, J.T., Schumacher, M.H., Rose, J.P., Raines, T.C., Politz, M.C., Copeland, M.F., and Pfleger, B.F. (2013). Production of medium chain length fatty alcohols from glucose in Escherichia coli. *Metabolic engineering* 20**,** 177-186.
